# The association of serum estradiol level with outcomes of clomiphene citrate/human menopausal gonadotropin ovarian stimulation for in vitro fertilization and embryo transfer

**DOI:** 10.1186/s12958-015-0109-x

**Published:** 2015-10-06

**Authors:** Xiao-Jin Zhang, Su-Ying Liu, Wei Fu, Xiao-Xi Sun

**Affiliations:** Obstetrics and Gynecology Hospital, Fudan University, Shanghai, 200090 China; Shanghai Ji Ai Genetic and IVF Institute, 588 Fangxie Road, Shanghai, 200011 China

**Keywords:** Clomiphene citrate, Human menopausal gonadotropin, IVF, Estradiol, Frozen-thawed embryo transfer

## Abstract

**Background:**

The purpose of this study was to test the hypothesis that estradiol (E_2_) level on day 3 may be associated with *in vitro* fertilization (IVF) outcomes.

**Methods:**

The records of patients who received clomiphene citrate 100 mg/day plus human menopausal gonadotropin 150 IU/day from day 3 and received frozen-thawed embryo transfers were reviewed. Patients were divided into three groups: group A (E_2_ ≤30 pg/ml), group B (30< E_2_ ≤50 pg/ml), and group C (E_2_ >50 pg/ml). A total of 1080 cycles from 941 patients were included.

**Results:**

The number of eggs and MII oocytes were less in group C than group A (both, *P* = 0.001). The embryo implantation (*P* = 0.006) and clinical pregnancy rates (*P* = 0.036) were lower in group C than group B, and the rates were similar between group A and B.

**Conclusion:**

Maintaining the serum E_2_ level from 30 to 50 pg/ml may result in a higher clinical pregnancy rate in IVF cycles.

## Introduction

Ovarian stimulation is an integral part of *in vitro* fertilization (IVF), and multiple oocytes are usually retrieved during a successful IVF cycle [1]. In recent years there has been an increasing trend towards using milder ovarian stimulation protocols to decrease the risk of ovarian hyperstimulation syndrome (OHSS) [2, 3]. In addition to decreasing the risk of OHSS, milder stimulation protocols can significantly decrease the physical and psychological stress of patients, increase patient convenience, reduce treatment costs, and reduce the risk of aneuploidy in human preimplantation embryos [4, 5].

Clomiphene citrate (CC) has been used for over 40 years as the primary drug for ovarian stimulation during IVF, and is the drug of choice for first line treatment of anovulatory dysfunction stemming from a variety of causes [6–8]. It is orally administered, has few side effects, is easily available, and is inexpensive [9]. In recent years the use of CC has increased because of the increased use of mild and mini ovarian stimulation protocols [10, 11].

As is well-known, CC is a competitive inhibitor of estradiol (E_2_) and can block the negative feedback of E_2_ by binding to estrogen receptors at the hypothalamic level, thus increasing the endogenous secretion of follicle stimulating hormone (FSH) [12]. CC is normally used from cycle day 3 onward. Ovarian function varies in the menstrual cycle, and in both long and short stimulation protocols E_2_ is routinely measured before the initiation of ovulation. For patients with a poor ovarian reserve, FSH should be measured before ovulation, and there is a relationship between FSH and E_2_: FSH decreases as E_2_ increases, and E_2_ is more stable than FSH. Thus, E_2_ may reflect ovarian function. The E_2_ level on cycle day 3 varies widely among patients, but it is not known if the E_2_ level on cycle day 3 has an effect on the outcomes after CC ovarian stimulation. Some studies, however, have suggested that E_2_ level early in the cycle is associated with IVF outcomes. An early study by Phelps et al. [13] found that an E_2_ level on day 4>75 pg/ml was associated with higher clinical pregnancy and delivery rates than a level ≤75 pg/ml. Similarly, Prasad et al. [14] reported that a higher E_2_ level on day 2 and on the day of trigger was associated with a higher pregnancy rate in women undergoing IVF.

Thus, the purpose of this study was to determine if E_2_ level on cycle day 3 is associated with IVF outcomes in women receiving CC ovarian stimulation. The findings may help to determine the optimal E_2_ level at which to initiate CC.

## Materials and methods

### Study subjects and protocol

The records of patients who underwent a CC plus human menopausal gonadotropin (hMG) protocol at the Shanghai Ji’ai Genetics and IVF Institute between October 2012 and September 2013 were retrospectively reviewed. Inclusion criteria for the study were: 1) Poor ovarian response after prior stimulation with a long or short protocol, and the number of collected oocytes was no higher than 3; or 2) Baseline FSH was greater than 10 IU/ml at the initiation of first ovulation. Exclusion criteria were: 1) Polycystic ovary syndrome (PCOS); 2) Prior ovarian response was adequate; 3) Ovarian cysts; 4) Endometriosis. This study was approved by the Institutional Review Board of the Shanghai Ji’ai Genetics and IVF Institute, and because of the retrospective nature the requirement of informed patient consent was waived.

Ovarian stimulation was carried out using CC (Clomiphene Citrate Tablets, Codal Synto Ltd., Limassol, Cyprus) in combination with hMG (Menotrophins for Injection, Lizhu Pharmaceutical, China). Per protocol, CC 100 mg/day was administrated orally as an extended regimen from cycle day 3 until induction of oocyte maturation, and hMG (150 IU/day) was administered during the same period. Monitoring included ultrasonography and measurement of serum concentrations of E_2_, progesterone, and luteinizing hormone (LH), and was usually begun on day 9 and continued every day or every other day according to the development of follicles. Maturation was triggered by administration of nafarelin acetate (Synarel, Pfizsurge, USA) 200 mg, a gonadotropin-releasing hormone agonist (GnRHa), when the diameter of the dominant follicle had reached 18 mm or more. Nafarelin was used as the trigger because nafarelin is convenient to use and patients can inject it themselves. In addition, when CC is used there is no pituitary down-regulation. Thus, nafarelin may induce an endogenous LH peak to promote the maturation of follicles. Oocytes were then retrieved after 34–35 h. After oocyte retrieval, IVF were performed according to sperm quality.

Embryo development was observed on day 3 after IVF. Good quality for day 3 embryos was defined as grades 1 and 2 according to Scott’s criteria [15]. Embryos with six cells or more were cryopreserved using vitrification as previously described [16, 17]. Briefly, vitrification was performed in 7.5 % dimethyl sulfoxide (DMSO)/7.5 % ethylene glycol (EG) for 2–3 min followed by incubation in 15 % DMSO/15 % EG for 45 s prior to loading on the vitrification carrier. The cryoprotectant was removed during warming by sequential washes in 0.25 M and 0.125 M sucrose in culture medium.

Natural or hormone replacement cycles were used according to the patient’s menstruation. Endometrial thickness and shape were observed by ultrasound from cycle day 10. All patients underwent frozen-thawed embryo transfer (FET) when the endometrial thickness reached 8 mm, and a total of 1–3 embryos were transferred per cycle. Luteal phase support was provided with progesterone 40 mg/d by intramuscular injection and dydrogesterone (Citicoline Sodium and Glucose Injection, Solvay Pharmaceutical) 20 mg/d orally. Beta-human chorionic gonadotropin (β-hCG) concentration was measured on day 14 after embryo transfer. Implantation was considered to have occurred when the β-hCG concentration was >200 mIU/ml. Ultrasound was performed 2 weeks later, and clinical pregnancy was defined as the presence of a fetal heartbeat.

### Statistical analysis

Continuous variables were expressed as median (IQR; interquartile range, range between the 25^th^ and 75^th^ percentile) due to skewed distributions, and the Kruskal-Wallis test was carried out to test differences among patients with the three E_2_ levels. Mann–Whitney *U* test was performed for post-hoc analysis of continuous data if any significance was revealed by the Kruskal-Wallis test. Categorical data were expressed as count (percentage), and tested by chi-square test. In order to control for the effects of age, body mass index (BMI), and E_2_ level at baseline, analysis of covariance (ANCOVA), Poisson regression, and logistic regression were implemented for continuous variables, count data (i.e., successful fertilization and successful cleavage), and cancelled cycles, respectively. Due to the assumptions of the parametric method, rank transformation was applied to scale outcomes with skewed distributions (e.g., FSH during ovulation stimulation and duration of stimulation) before multivariable analysis. A value of *P* < 0.05 was considered to indicate statistical significance. If post-hoc testing was necessary, Bonferroni correction was performed and the significance level was adjusted to 0.017 (0.05/3). All analyses were performed with IBM SPSS statistical software for Windows (Version 22.0. IBM Corp., Armonk, NY, USA).

## Results

### Study population

A total of 1080 cycles of 941 patients were included in this study. Data were classified into three groups according to E_2_ level measured on day 3 during ovulation stimulation: group A (E_2_ ≤30 pg/ml, 316 cycles), group B (30< E_2_ ≤50 pg/ml, 451 cycles), and group C (E_2_ >50 pg/ml, 313 cycles). As shown in Table 1, the three groups were similar with respect to duration of infertility, ovarian stimulation cycles, number of embryos transferred, and baseline FSH level. However, patients in group C were older than those in group A (*P* = 0.015), and had a lower BMI than patients in the other two groups (*P* = 0.010 compared with group A; *P* = 0.004 compared with group B). The baseline E_2_ level of patients in group A was significantly lower than that in group B and C (both, *P* < 0.001).

**Table 1:** Baseline characteristics of the three study groups

	Group A:	Group B:	Group C:	*P*
	E_2_ ≤ 30 pg/ml	30 < E_2_ ≤ 50 pg/ml	E_2_ > 50 pg/ml	
Number of cycles	316	451	313	
Age, years	34 (31, 38)	35 (31, 39)	35 (31, 40)*	0.049
Body mass index, kg/m^2^	21.2 (19.9, 23.3)	21.4 (20, 23.1)	20.8 (19.5, 22.2)* **	0.008
Duration of infertility, years	5 (3, 9)	5 (3, 8)	5 (3, 8)	0.809
Number of past ovarian stimulation cycles	2 (1, 3)	2 (1, 3)	2 (1, 2)	0.487
Number of past embryo transfers	2 (1, 3)	2 (1, 2)	1 (1, 2)	0.146
Baseline FSH, IU/L	8.6 (7.1, 10.7)	8.7 (7.1, 11.1)	8.9 (7.3, 11.8)	0.353
Baseline E_2_, pg/mL	33 (24, 45)	38.5 (29, 50.5)*	48 (33, 62)* **	<0.001

Data are presented as median (interquartile range), and examined by Kruskal-Wallis test

*FSH* follicle-stimulating hormone

*Indicates significant difference between group A and given group, *P* < 0.017

**Indicates significant difference between group B and group C, *P* < 0.017

### Ovarian stimulation and fertilization

FSH level during ovulation stimulation, E_2_ level on HCG day, number of embryos, and good embryos were similar across the three groups. The fertilization rate, cleavage rate, and percentage of patients with canceled cycles were also similar (Table 2). However, the duration of stimulation, gonadotropin level, and the number of eggs and mature oocytes (MII) were different among the three groups. Compared with group A, the duration of stimulation was shorter and the amount of gonadotropin used was less in group B (both, *P* ≤ 0.004) and C (both, *P* ≤ 0.001), and the duration of stimulation was shortest in group C (*P* = 0.001 compared with group B). The number of eggs and MII oocytes were also less in group C than group A (both, *P* = 0.001) (Table 2). Results of the multivariable analysis were consistent, except for the comparison of FSH during ovulation stimulation; although a significant difference of FSH between the three groups was revealed by ANCOVA, no pair-wise differences were found after Bonferroni correction.

**Table 2:** Serum E_2_ levels and ovarian stimulation and fertilization outcomes

	Group A:	Group B:	Group C:	*P*	*P*_*adj*_^e^
	E_2_ ≤30 pg/ml	30< E_2_ ≤50 pg/ml	E_2_ >50 pg/ml		
Number of cycles	316	451	313		
FSH during ovulation stimulation, IU/L^a^	9.5 (7.5, 11.8)	8.9 (7.5, 10.6)	9 (7.7, 11)	0.098	0.047
Duration of stimulation, days^a^	9 (8, 10)	8 (7, 10)*	8 (7, 9)* **	<0.001	<0.001
Gonadotropin, ampules	16 (12.5, 18)	14 (10, 18)*	14 (10.8, 17.5)*	<0.001	<0.001
E_2_ at HCG day, pg/ml^a^	2216 (1391, 3319)	2150.5 (1406, 3294)	2179.5 (1337.5, 3223)	0.991	0.976
Number of eggs^a^	6 (3, 9)	5 (3, 8)	5 (2, 7)*	0.004	0.031
Number of mature oocytes (MII)^a^	5 (3, 8)	4 (2, 7)	4 (2, 6)*	0.003	0.032
Number of embryos^a^	2 (1, 4)	2 (1, 4)	2 (1, 4)	0.340	0.771
Number of good quality embryos^a^	2 (1, 4)	2 (1, 3)	2 (1, 3)	0.440	0.638
Successful fertilization^b,c^	1470 (82.7)	1968 (84.2)	1261 (82.9)	0.375	0.756
Successful cleavage^b,d^	1432 (97.4)	1917 (97.4)	1217 (96.5)	0.257	0.995
Cancelled cycles^b^	46 (14.6)	55 (12.2)	52 (16.6)	0.221	0.290 (Group B), 0.766 (Group C)

*E*_*2*_ estradiol, *HCG* human chorionic gonadotropin

Continuous data are presented as median (interquartile range), and examined by Kruskal-Wallis^a^ test. Categorical data are expressed as n (%) and tested by Chi-square^b^ test

^c^Fertilization rate was calculated as: total number of fertilized oocytes/total number of mature oocytes

^d^Cleavage rate was calculated as: total number of cleaved oocytes/total number of fertilized oocytes

^e^Rank transformation was applied to continuous data, and tested by analysis of covariance adjusting for age, body mass index (BMI), and baseline E_2_ level. Successful fertilization and cleavage rate were tested by Poisson regression adjusting for age, BMI, and baseline E_2_ level. Probability of cancelled cycle was examined by logistic regression adjusting for age, BMI, and baseline E_2_ level

*Indicates significant difference between group A and given group, *P* < 0.017

*Indicates significant difference between group B and group C, *P* < 0.017

### FET results

Except for the embryo implantation rate and ongoing clinical pregnancy rate, FET outcomes were similar between the groups (Table 3). Although patients in group B seemed to have the highest implantation and clinical pregnancy rates, only the differences between group B and group C reached statistical significance; higher probabilities of embryo implantation (*P* = 0.023) and ongoing pregnancy (*P* = 0.036) were seen in group B as compared with group C.

**Table 3:** Serum E_2_ levels and frozen embryo transfer (FET) outcomes

	Group A:	Group B:	Group C:	*P*	*P*_*adj*_^d^
	E_2_ ≤ 30 pg/ml	30 < E_2_ ≤ 50 pg/ml	E_2_ > 50 pg/ml		
Number of transfer cycles	241	350	225		
Number of transferred embryos^a^	2 (2, 3)	2 (2, 3)	2 (2, 3)	0.574	0.510
Number of transferred good quality embryos^a^	2 (1, 2)	2 (1, 2)	2 (1, 2)	0.366	0.431
Endometrial thickness, mm^a^	10 (9, 11)	10 (9, 11)	10 (9, 11)	0.543	0.383
Successful embryo implantation^b,c^	115 (22.0)	195 (25.1)	93 (18.6)*	0.023	-
Ongoing clinical pregnancy^b^	94 (29.9)	154 (34.1)	78 (25.1)*	0.027	0.552 (Group A)
					0.036 (Group B)
Abortion^b^	9 (2.8)	18 (4)	16 (5.1)	0.349	0.198 (Group A)
					0.520 (Group B)
Ectopic pregnancy^b^	3 (0.9)	6 (1.3)	1 (0.3)	0.357	0.410 (Group A)
					0.213 (Group B)

Dash indicates statistical analysis with adjustment was unavailable as there were second-hand data used for analysis

Continuous data are presented as median (interquartile range) and examined by Kruskal-Wallis^a^ test. Categorical data are expressed as n (%) and tested by chi-square test^b^

^c^Embryo implantation rate was calculated as: total number of embryos which are implanted/total number of embryos transferred

^d^Rank transformation was applied to continuous data, and tested by analysis of covariance adjusting for age, body mass index (BMI), and baseline E_2_ level. Probabilities of ongoing clinical pregnancy, abortion, and ectopic pregnancy were examined by logistic regression adjusting for age, BMI, and baseline E_2_ level

*Indicates significant difference between group B and group C, *P* < 0.017; chi-square test with Bonferroni correction

## Discussion

The results of this study showed that patients with a lower E_2_ level on day 3 required a greater amount of gonadotropin and longer duration of stimulation, though other measures were similar between the groups. Most importantly, the embryo implantation and clinical pregnancy rates in group B (30< E_2_ ≤50 pg/ml) were higher than in group C (E_2_ >50 pg/ml), and similar to that in group A (E_2_ ≤30 pg/ml).

In this study there are a number of reasons for the classification of E_2_ level, and for examining E_2_ level on day 3. Estrogen has a normal distribution, and an estrogen of 30–50 pg/ml is found in about 90 % of subjects. We have also clinically observed that 50 pg/ml is an upper limit of normal for E_2_. Furthermore, at our hospital a chemiluminescence method was used to detect the blood estrogen, and the value of 30 pg/ml is the lower limit of detection of chemiluminescent methods (values <30 pg/ml may not be measured precisely). With respect to classifying E_2_ on day 3, first available studies focus on E_2_ on the day of HCG use, or several days after Gn administration, and no studies have been conducted to investigate the relationship between E_2_ before Gn therapy and outcomes. Second, CC + hMG was administered since day 3 of the menstrual cycle when FSH and E_2_ are routinely measured to evaluate ovarian function and guide Gn therapy. In clinical practice, we found that E_2_ was related to FSH and the central feedback of E_2_ could directly affect FSH and then influence the response to ovulation, which is usually neglected. Most investigators have focused on FSH. Third, during ovulation E_2_ may be used to reflect the ovarian response. However, E_2_ before therapy may also reflect the ovarian reserve, and early detection of E_2_ may be helpful to guide clinical therapy. E_2_ is an important hormone of the hypothalamic-pituitary-ovarian axis, and positive feedback of E_2_ in the luteal phase may induce FSH release and recruitment of follicles.

One of the major challenges of assisted reproduction technologies (ART) is developing effective approaches to obtain good quality embryos for transfer. Thus, improving the stimulation protocol is an important area of IVF research. CC is now wildly used in mini and mild ovarian stimulation protocols, and provides advantages over other medications [2, 18, 19]. Clomiphene compounds include enclomiphene and zuclomiphene. Enclomiphene may affect the hypothalamus as an estrogen antagonist, and thus blocks both the negative and positive feedback of estrogen, and the result of these effects is alterations in GnRH pulsatility. Zucolmiphene may weakly affect the pituitary as an estrogen agonist, and it can slightly increase the sensitivity of the pituitary gland to GnRH [2]. The overall effect of clomiphene compounds is to trigger the release of both FSH and LH from the anterior pituitary. FSH stimulates the development of follicles, which then release E_2_. Once serum E_2_ reaches a critical level, it triggers the release of LH and ovulation. GnRH agonists and antagonists have been used to prevent this, but in recent years it has been noted that the administration of CC until the day before HCG with prevent the premature LH surge [2, 19]. This is because enclomiphene acts as an estrogen antagonist at the level of the hypothalamus, and not only blocks the negative feedback of E_2_, but also its positive feedback resulting in blockage of the LH surge [20]. Although the premature LH surge is blocked by CC, the LH level is adequate for the development of follicles to the late phase, and this method provides better results than an agonist and antagonist [21]. Interestingly, Ye et al. [22] found that luteal E_2_ pre-treatment before a GnRH antagonist protocol significantly increased the serum LH level and rate of premature LH surge, but had no significant effect on implantation, clinical pregnancy, live birth, and early pregnancy loss rates as compared with a long GnRH agonist protocol.

FSH level is typically tested at the beginning of stimulation [23, 24], and the importance of E_2_ level is frequently overlooked. However, a number of studies have examined the effect of E_2_ level and IVF outcomes. Phelps et al. [13] examined patients undergoing IVF with luteal phase leuprolide acetate and found that an E_2_ level on day 4>75 pg/ml was associated with clinical pregnancy and delivery rates of 42 and 32 %, respectively, which were significantly higher than rates of 9 and 6.8 %, respectively, when the day 4 E_2_ level was ≤75 pg/ml. Prasad et al. [14] reported that E_2_ levels on day 2 and on the day of trigger in women undergoing IVF were higher in those that became pregnant than those that did not become pregnant (31.9 ± 12.6 pg/ml and 1996.46 ± 1252.36 pg/ml vs. 27.6 ± 12.3 pg/ml and 1525.1 ± 1116.42 pg/ml, respectively). DiMattina et al. [25] examined patients undergoing natural cycle IVF and found that when the E_2_ level on the day of hCG was <101 pg/ml no clinical pregnancies occurred.

In a study of 2995 consecutive IVF cycles in 1889 patients with non-donor oocyte retrieval and FET, Imudia et al. [26] found that serum E_2_ above the 90^th^ percentile on the day of hCG administration was associated with a significantly lower fertilization rate as compared with patients with a serum E_2_ below the 90^th^ percentile (68.6 ± 20 % vs. 71.6 ± 21 %, *P* = 0.02); but the E_2_ level did not impact embryo development, implantation, or clinical pregnancy and spontaneous miscarriage rates. Kondapalli et al. [27] examined 1712 IVF cycles, and reported that a >10 % decrease in E_2_ level after hCG administration was associated with 40–50 % reduction in the clinical pregnancy and live birth rates, and a post-hCG E_2_ plateau ± 10 % lowered the clinical pregnancy and live birth rates by >25 %.

The standard initial ovarian stimulation protocol used at our center consists of luteal phase GnRHa down-regulation followed by administration of exogenous gonadotropins. This method has been guided by the increased number of oocytes and greater pregnancy rate obtained with this combination, as well as the benefits of better scheduling and lower cancellation rates due to premature LH surges following pituitary desensitization [28]. However, patients who fail or respond poorly to standard controlled ovarian stimulation protocols often continue to respond poorly in subsequent cycles. Current evidence suggests that increasing the dose of exogenous gonadotropins is of limited value for these patients [29]. In patients with a poor response to gonadotropins, Kutlusoy et al. [30] found that the clinical pregnancy rate was significantly higher in patients that received luteal phase support of 2 mg E_2_ plus progesterone than in those that received progesterone only (37 % vs. 12 %, respectively). Elassar et al. [31] showed that when low responders received a higher dose of gonadotropins in a successive cycle, the magnitude and pattern of the ovarian response remained unchanged. Similarly, van Hooff et al. [32] reported that doubling the hMG dose in the course of an IVF cycle is not effective in enhancing ovarian response. These findings suggest that some patients appear to respond more favorably to their endogenous gonadotropins [33].

There are limitations of this study that need to be considered. First is the retrospective nature of the analysis. In addition, the study was performed at a single center and examined the results of a single stimulation protocol. Both natural and hormone replacement cycles were included in the analysis, and the type of cycle may have an effect on IVF outcomes. Other factors associated with IVF outcomes such as anti- Müllerian hormone and progesterone levels were not examined [34, 35]. Lastly, at our center the protocol for ovulation induction is determined according to the ovarian reserve, and not selected subjectively. The patients in the present study had poor ovarian reserve or were older, and had poor responses to ovulation stimulation with other protocols. Thus, the patients may be considered a group in whom it is difficult to induce ovulation.

## Conclusion

Serum E_2_ level on day 3 can affect the duration of stimulation and amount of gonadotropins used, and a level of 30–50 pg/ml is associated with a higher clinical pregnancy rate than levels above this range.
